# Supplementary material for: Trends of Medical Service Utilization for Tinnitus: Analysis Using 2010–2018 Health Insurance Review and Assessment Service National Patient Sample Data
Source: Healthcare (Basel). 2022 Aug 16;10(8):1547. doi: 10.3390/healthcare10081547 (PMC9408172; doi:10.3390/healthcare10081547)
Supplement: Supplementary file 1 [file healthcare-10-01547-s001.zip › healthcare-1838510-supplementary.pdf]

Supplementary Table S1. Annual average KRW-USD exchange rate and price level of health expenditure.

Year	KRW/USD	Price level
2010	1,156.00	0.9345
2011	1,107.99	0.9510
2012	1,126.76	0.9597
2013	1,095.04	0.9631
2014	1,053.12	0.9699
2015	1,131.52	0.9820
2016	1,160.41	0.9918
2017	1,130.48	1.0005
**2018**	**1,100.58**	**1.0000**

This information is available here: Korean Statistical Information Service (http://kosis.kr).

The price level of health expenditure is adjusted as of the year 2018.

Supplementary Table S2. Basic characteristics of medical usage.

Category		Total claims (2010-2018)		WM (2010-2018)		KM (2010-2018)	
Type of visit	Outpatient	309,842	99.92	202,239	99.89	107,603	99.97
	Inpatient	256	0.08	224	0.11	32	0.03
Medical institution	Tertiary hospital/general hospital/hospital	52,501	16.93	52,279	25.82	222	0.21
	Clinic	150,170	48.43	150,170	74.17	-	-
	KM hospital	3,965	1.28	14	0.01	3,951	3.67
	KM clinic	103,462	33.36	-	-	103,462	96.12

WM: Western Medicine; KM: Korean Medicine.

All expenditures were converted based on the annual average exchange rate (KRW/USD) and the price is adjusted as of health expenditure price level for the year 2018. (See Table 1)

Supplementary Table S3. High frequency care for tinnitus in WM.

		WM outpatients		
		Total claims	Average expenditure per claim	Average expenditure per patient
Auditory function test	1-1. Test by pure tone audiometer	66,175	10.32	16.41
	1-2. Hearing test (test by impedance audiometry)	38,269	7.53	10.22
	1-3. Speech audiometry	20,692	14.36	16.32
	1-4. Otoacoustic emission test	9,929	15.41	21.26
	1-5. Brain evoked potential test	3,064	39.31	39.99
	1-6. Auditory brainstem response threshold test	1,895	88.64	91.09
	1-7. Electrocochleography	594	38.45	39.72
	1-8. Tympanoscopy	583	23.24	29.97
Tests (excluding blood test)-Auditory function test x		17,584	11.17	25.85
Injection		15,722	1.30	3.08
Treatment and procedures		37,445	4.44	10.41
Blood test/urinalysis		36,345	2.50	35.53
Management/supervision		24,692	0.56	1.63
Others		48,615	1.87	10.43

WM: Western Medicine
All expenditures were converted based on the annual average exchange rate (KRW/USD) and the price is adjusted as of health expenditure price level of year 2018. (See Table 1)

Supplementary Table S4. High frequency care for tinnitus in KM.

	KM Outpatients		
	Total claims	Average expenditure per claim	Average expenditure per patient
Acupuncture	203,723	3.72	55.15
Electroacupuncture stimulation	12,874	3.84	23.47
Cupping (dry)	18,370	3.45	21.51
Cupping (bloodletting)	11,158	5.93	30.99
Moxibustion (direct)	28,462	2.31	17.23
Moxibustion (indirect)	3,197	5.67	33.21
Hot/cold meridian therapy – infrared therapy	30,581	0.84	5.70
Hot/cold meridian therapy – hot pack therapy	7,119	0.84	5.01
Herbal formulation	10,811	0.45	2.28
KM tests	2,520	3.66	4.25
Other treatment	23,344	5.91	13.31

KM: Korean Medicine.

All expenditures were converted based on the annual average exchange rate (KRW/USD) and the price is adjusted as of health expenditure price level for the year 2018. (See Table 1)

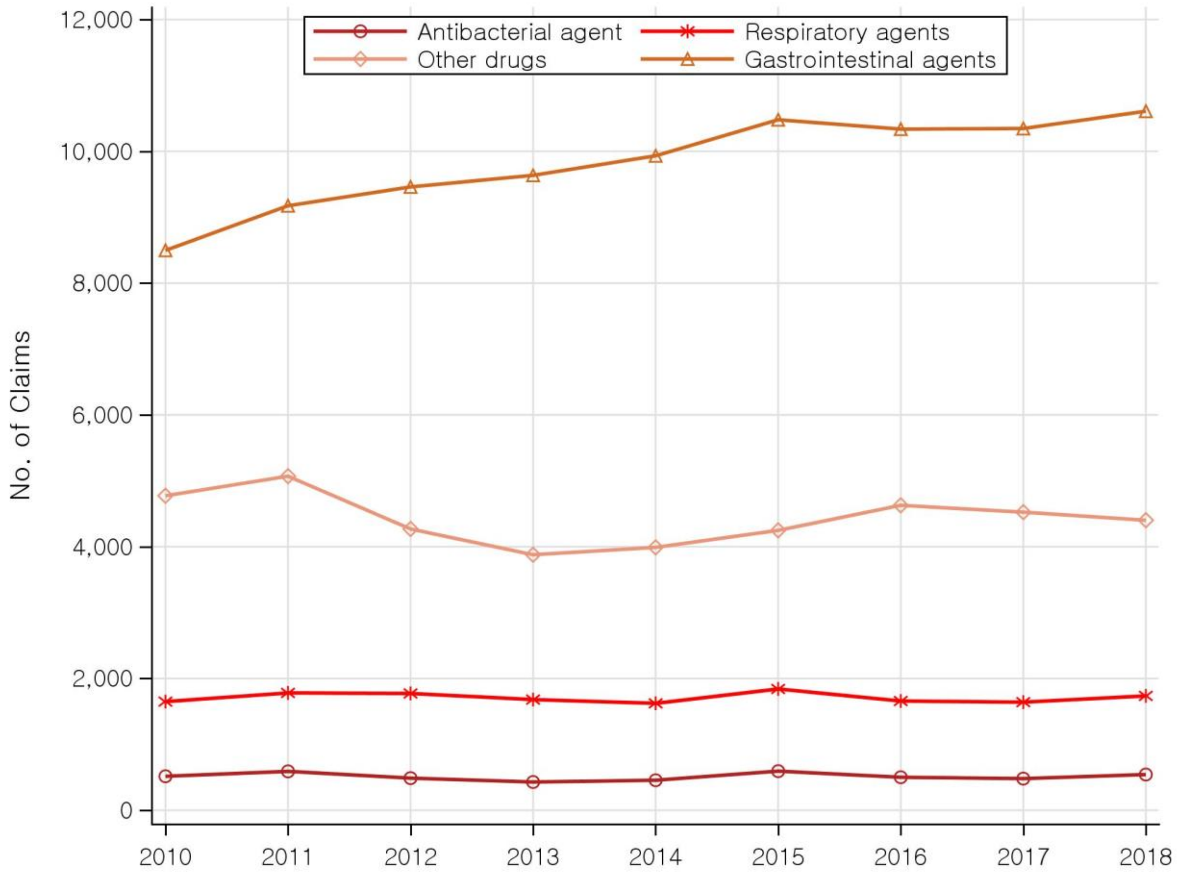

Supplementary Figure S1. Yearly trend in prescription of other drugs.
